# Differences in Body Composition in Older People from Two Regions of Mexico: Implications for Diagnoses of Sarcopenia and Sarcopenic Obesity

**DOI:** 10.1155/2018/7538625

**Published:** 2018-07-09

**Authors:** Diana Beatriz Rangel Peniche, Heliodoro Alemán Mateo, Ma. de los Angeles Aguilera Barreiro, Roxana E. Ruiz Valenzuela, Maribel Ramírez-Torres, Rene Urquidez-Romero

**Affiliations:** ^1^Facultad de Ciencias Naturales, Universidad Autónoma de Querétaro, Campus Juriquilla. Boulevard de las Ciencias S/N, C.P. 76230 Querétaro, QRO, Mexico; ^2^Departamento de Nutrición y Metabolismo, Coordinación de Nutrición, Centro de Investigación en Alimentación y Desarrollo, A.C. Carretera a La Victoria km 0.6, Apdo. Postal 1735, C.P. 83304 Hermosillo, SON, Mexico; ^3^Departamento de Ciencias de la Salud, Universidad Iberoamericana Ciudad de México-Tijuana, Av. Centro Universitario 2501, Playas de Tijuana, C.P. 22500 Tijuana, BC, Mexico; ^4^Departamento de Ciencias de la Salud, Instituto de Ciencias Biomédicas, Universidad Autónoma de Ciudad Juárez, Ave. Plutarco Elías Calles #1210, Fovissste Chamizal, C.P. 32310 Ciudad Juárez, CHIH, Mexico

## Abstract

**Background:**

Mexico is a country that is rich in ethnicity and cultural diversity, divided into three well-defined socioeconomic, ecological, and epidemiological areas. However, we do not know the influence that these factors may have on body composition. Therefore, this study was designed to assess body composition and compare appendicular skeletal muscle mass (ASM) in older people from two areas of the country.

**Methods:**

This is a cross-sectional study that included 430 subjects ≥60 years of age from northwestern and central Mexico. Body composition, including ASM, was measured by dual-energy X-ray absorptiometry, while anthropometry, handgrip strength, demographic variables, health status/chronic conditions, and energy expenditure data were all included.

**Results:**

Men and women from the northwestern region had 5.9 kg and 3.8 kg more body fat, respectively, and 3.9 kg more as a group than their counterparts from central Mexico (*p* ≤ 0.0001). While there were no significant differences across gender or region in terms of ASM, the older subjects from central Mexico had a significantly higher ASM index (ASMI) than the sample from the northwest. When ASM was adjusted for age, body weight, height, health status/chronic conditions, estimated energy expenditure, and demographic variables, the subjects from central Mexico had significantly higher adjusted mean values of ASM and ASMI than their counterparts from the northwest.

**Conclusion:**

Older people from two regions of Mexico had significantly different estimates of body composition. Our findings highlight the importance of regionalizing estimates of ASM and ASMI if they are to be used for diagnostic purposes. It is also important to emphasize that appendicular skeletal muscle mass, or the ASM index, should be adjusted for other associated biological variables.

## 1. Introduction

Recently, age-related changes in body composition, including a decrease in fat-free mass [1, 2], an increase in body fat compartment [2], and the loss of skeletal muscle [3], have been shown to be strongly associated with a greater risk of functional disability, including impaired physical performance [4–6]. Therefore, assessing elderly populations using the most precise and accurate body composition methods should be a priority for achieving better clinical diagnoses. Also, body fat mass (BFM) divided for the height can be an adequate marker of overweightness and obesity in American populations [7], while total or appendicular skeletal muscle (ASM) divided by height squared (i.e., the ASM index or ASMI) can be used as markers of sarcopenia [8]. These findings demonstrate the need to adjust body composition parameters for better clinical interpretation and comparison and underscore the need to consider age, gender, ethnicity, and body weight, and height when focusing on the skeletal muscle component [3, 9]. Also, other nonbiological factors must be included to obtain more specific data for different age groups, lifestyles, and ethnic groups, since these parameters may limit the usefulness of such data for purposes of universal diagnoses.

With respect to ethnicity, older mestizo people from northwestern Mexico were found to have significantly lower ASM and higher BFM and truncal fat than older Caucasian and Afro-American men and women with the same body mass index (BMI) [10]. The effect of ethnicity on skeletal muscle as an index is well recognized in different age groups [11]; for example, the ASMI of young Asian men and women is more similar to that of young Mexican men and women, but when compared to other ethnic groups, Mexican ASMI are significantly lower [12]. To date, few studies have explored the influence of lifestyle factors on skeletal muscle. Traditionally, age, height, body weight, gender, and ethnicity have been considered as the main determinants of the skeletal muscle component [3, 9]. The importance of skeletal muscle in elderly populations lies in the fact that low skeletal muscle mass is strongly associated with physical disability. Recently, Tyrovolas et al. [13] showed that lower levels of education and wealth, high body fat, consumption of alcoholic beverages, and chronic conditions were all significantly associated with lower skeletal muscle index.

The ethnic diversity of the Mexican population ranks among the top eight countries in the world. For Mexican public health and nutrition policies, the country has been divided into three well-defined socioeconomic, ecological, and epidemiological areas [14], but at the national level little is known about how ethnic, environmental, or lifestyle factors affect body composition parameters, especially BFM [15] and skeletal muscle mass. In relation to these three well-defined areas [14] and based on national-level anthropometric data, we assume that people from the northwest weigh more and are taller than people from central Mexico. The primary objective of this study, then, was to measure body composition by dual-energy X-ray absorptiometry (DXA); the second was to compare appendicular skeletal muscle mass in older people from two distinct areas of the country. We found that older mestizo people from the northwest had higher ASM and ASMI than mestizo men and women from central Mexico; however, adjustments for traditional variables and other skeletal muscle-related lifestyle variables including body fat and data on energy expenditures changed the results considerably.

## 2. Methods

This is a cross-sectional study based on a nonrandomized sample of 430 apparently healthy older adults from two regions of Mexico. All data for both samples were gathered using the same protocol,* that is*, anthropometry, body composition, and strength measurements. The protocol was approved by the Ethics Committee of each participating institution.

### 2.1. Subjects

The study included men and women (aged 60 to 83 years) from the municipality of Queretaro in central Mexico and subjects living in Hermosillo, Sonora, in northwestern Mexico. All participants were in free-living conditions. Some spent time at day institutions performing leisure activities; others had part-time jobs, and some simply spent time in their homes in mostly sedentary activities. Institutions for the elderly were visited to invite potential participants to enroll in the study. Other subjects were invited through home visits or by phone. All potential subjects received a complete explanation of the protocol and were invited to the Nutrition Clinic at the Autonomous University of Queretaro and to the Laboratory of Body Composition and Functionality, Coordination of Nutrition, and Research Center for Food and Development in Hermosillo, where the study procedures were explained in detail. Once their questions had been answered, they were asked to sign an informed consent form.

All volunteers underwent a clinical assessment that included anthropometry and body composition measurements by DXA. According to their clinical histories, some subjects were free of disease, but others had chronic diseases that were controlled by medication and laboratory tests. In general, all volunteers were free of medications that might affect their hydration status and body composition. Use of medicines was evaluated only by self-reporting, while health status was assessed clinically, including some laboratory determinations. According to their self-reports, subjects had stable body weight over the previous three months, were physically independent according to the Katz [16] and Lawton and Brody scales [17], and were free of cognitive problems according to Pfeiffer's scale [18] and the Mini-Mental State Examination modified by Icaza and Albala [19]. It is important to note that the regions included form part of the four regions classified in Mexico's National Health and Nutrition Survey (ENSANUT, 2012). They are well characterized by shared geographical and socioeconomic characteristics [20]. This regional scheme has been used in epidemiological studies to make comparisons within the country.

### 2.2. Measurements

Bone-free lean tissue and other body composition data, such as body fat mass compartment, truncal fat, and the ASM component, were estimated by DXA using the QDR-4500W1 Hologic Explorer (Hologic Inc., Waltham, MA). All measurements were made under fasting conditions with subjects wearing only a disposable gown, without metal accessories (e.g., watches, belts, and earrings), and after emptying the bladder. For bone-free lean tissue, or lean tissue, DXA utilizes the placement of standard cut-lines to assess the arms, legs, and trunk. The placement of cut-lines was according to the guidelines defined and recommended by Heymsfield et al. (1990) to estimate ASM as the sum of lean tissues in the arms and legs [21]. The use of DXA to estimate ASM (kg) is based on the assumption that lean tissue in the arms and legs represents limb skeletal muscle mass and that this represents 75% of total skeletal muscle mass [22]. Also, using the DXA-report, we obtained fat-free mass (FFM) (kg) as the sum of total lean tissue (TLT) (kg) plus total bone mineral content (TBMC, kg), according to the assumptions of a two-compartment model [23]. ASM, FFM, and BFM were divided by height squared (m^2^) to obtain three indices: ASMI (kg/m^2^), FFMI (kg/m^2^), and BFMI (kg/m^2^). All DXA measurements were performed by trained personnel, and all DXA scans were edited by the same standardized researcher in order to define ASM based on the placement of the cut-lines for the recommended regions of interest (ROI) [21]. Calibration was performed daily before taking measurements, following the procedure recommended by the supplier.

Body weight (kg) was measured using similar electronic scales (capacity 220 kg), while for standing height (cm) stadiometers with a precision of 20-205 cm ± 5 mm were utilized. Body mass index was derived (BMI, kg/m^2^). Waist and hip circumferences were measured using a metallic Rosscraft Anthrotape. All measurements were performed in triplicate and mean values were calculated and used.

Muscle strength (kg) in subjects from both regions was measured by handgrip strength dynamometry using a manual Takei Smedley dynamometer (Takei Scientific Instruments Co., Ltd., Niigata, Japan; measure range: 5-100 kg). In the northwest, the handgrip strength test was performed with subjects sitting on chairs with their arm resting on a flat surface to avoid involving other muscles in the test. For the subjects in central Mexico, in contrast, this parameter was measured with subjects standing. Participants exerted maximal force with arms hanging on both sides and elbows fully extended. Three measurements were recorded for each side and the maximum value was used for analysis.

*Covariates*. Most covariates were derived from anthropometry (body weight, kg; height, m; BMI, kg/m^2^; waist and hip circumference, cm) and body composition measurements (total body mass, kg; body fat mass, kg; truncal fat, kg; fat-free mass kg; ASM, kg; total bone mineral content, kg). Demographic data such as age in years, gender (male and female), marital and employment status, and educational level were also recorded. The latter was classified according to Mexico's educational system, including technical careers. Toxicities such as smoking and alcohol consumption were registered. Other variables related to health status/chronic conditions, such as hypertension, type 2 diabetes, hypothyroidism, osteopenia and osteoporosis (yes/no), and other diseases, were all self-reported and then registered in subjects' clinical histories. Total and resting energy expenditures (kcal/day) were estimated by age and Latin American-specific published predictive equations [24].

### 2.3. Data Analysis

To explore the influence of geographic region on body composition compartments and indices in relation to two areas—central and northwestern Mexico—we tested the following hypothesis: older mestizo people from northwestern Mexico have higher ASM and ASMI than mestizo men and women from the central region after adjusting for body weight and height, lifestyle (sociodemographic) variables, and other factors, such as data on body fat and energy expenditure. This hypothesis was tested by a *t*-test and a GML ANOVA using the statistical program NCSS 2001 (Number Cruncher Statistical System for Windows, Kaysville, UT, USA) and Fisher's LSD multiple comparison test to make pairwise comparisons among means. Before conducting the GLM ANOVA test, univariate and multivariate analyses by regression procedures were run in order to adjust only for significant predictor variables of ASM, ASMI, and BFM and BFMI in the models obtained (see Tables 2 and 3). These analyses were performed using STATA (version 11.0; Stata Corp, College Station, TX, USA). For all tests, statistical significance was set at* p* ≤ 0.05.

## 3. Results

### 3.1. Basic Anthropometry and Body Composition Data of Participants by Gender and Region

Overall data on age, anthropometric characteristics, and body composition are summarized in Table 1. Northwestern women, as well as men and women as a group, were older (*p* < 0.0001) than their counterparts in central Mexico. Also, the men and women from the northwest, both independently and as a group, weighed 8.2 kg, 6 kg, and 7.3 kg, respectively, more than their counterparts and were taller (*p* ≤ 0.0001) with significantly higher BMI than the older people in central Mexico. The men from the northwest and the women from central Mexico had significantly higher waist circumference values than their counterparts.

In relation to body composition compartments, the men and women from the northwest had higher BFM, both separately and as a group, approximately 5.9 kg, 3.8 kg, and 3.9 kg, respectively, more BFM than the older subjects in central Mexico (*p* ≤ 0.0001). Also, subjects from the northwest were fatter, according to the BFM divided by height squared, that is, the BFMI, than their counterparts in central Mexico (*p* ≤ 0.05). They also had significantly higher values for truncal fat by DXA (Table 1). In general, TLT and TBMC and, therefore, FFM were all higher in the older people from the northwest than their counterparts, though Table 1 shows a clear effect of gender and region on TBMC. Only older women, and men and women as a group, in the northwest had significantly higher TLT and FFM values than their counterparts in central Mexico. For FFM, after dividing for height squared, the FFMI was consistently higher in older people from central Mexico (*p* ≤ 0.0001). There were no significant differences between gender and region in ASM, but ASM divided by height squared, the ASMI, and the FFMI were significantly higher in men and women from central Mexico, both separately and as a group. Handgrip strength was higher in men and women subjects in central Mexico but was only significant for women.

### 3.2. Association of Potential and Independent Predictors of Body Composition Compartments and Components and Their Indices in Older People

A univariate analysis was run to explore possible statistical associations among BFM, BFMI, ASM, and ASMI, as dependent variables, with various demographic, health status, anthropometric, and body composition parameters as independent variables. Results of the significant associations (*p* ≤ 0.2) in men and women separately, and as groups by region, are shown in Tables 2 and 3. After this analysis, a multiple regression analysis was performed, which found significant contributions (*p* ≤ 0.05) of the independent predictors to each one of the aforementioned dependent variables or models (Tables 2 and 3).

### 3.3. Effect of Region on Body Composition Estimates and Their Respective Indices in Older People

After the multiple regression analysis, a GLM ANOVA was run. Results showed the opposite results, as presented in Table 1. After adjusting for certain covariates (Table 4), the women from central Mexico proved to be somewhat fatter, with significantly higher mean BFM and BFMI values than the older women from the northwest. The main BFM and BFMI values in men, and men and women as a group, were quite similar between the groups of men and regions.

With respect to skeletal muscle, the adjusted mean ASM values were approximately 1 kg higher in men and women separately and as a group in the older subjects from central Mexico compared to their counterparts. This analysis shows that men and women from this region, both separately and as a group, had higher ASM values after adjusting for certain associated anthropometric, body composition, and health status/chronic conditions and demographic variables (Table 4) previously selected by multiple regression analysis as independent predictors of ASM (Table 2). Both adjusted and nonadjusted mean ASMI values in men and women from central Mexico, both separately and as a group, were higher than their counterparts (*p* < 0.0001) (Table 4). This analysis confirms the importance of adjusting ASM by height squared and suggests that certain demographic, health status, or chronic conditions and anthropometric and body composition variables should be taken into account in order to adequately adjust ASMI in populations with significant differences in height and body weight.

## 4. Discussion

This study analyzed significant differences in anthropometry and body composition parameters between older people from two regions of Mexico which are clearly characterized by certain shared geographic and socioeconomic characteristics [14, 20]. It also tested the influence of several demographic and health status/chronic conditions, estimated energy expenditure, and anthropometric and body composition variables on BFM and BFMI and ASM and ASMI in older people in an approach that has not been explored previously. Our results not only confirm that differences related to regionalization exist but also point out the importance of obtaining regional data on body composition and its indices in order to make reliable diagnoses of sarcopenia or sarcopenic obesity in older populations. Our findings further highlight the importance of adjusting ASM for some nonbiological covariates, as well as for age, weight, height, and gender [3, 9], to detect significant differences in the body composition-related muscularity index.

Recent evidence underscores the importance of maintaining skeletal muscle mass as a key factor in optimizing the health of older people [25]. The loss of skeletal muscle is strongly associated with increased risks of morbidity, physical disability, frailty [4–6, 26, 27], and mortality [27]. This process begins around the fourth decade of life and advances at a rate of approximately 0.8% per year. It is now recognized as an important component of sarcopenia syndrome [28]. But, in addition to age, the skeletal muscle component also depends on body weight and height, gender, and ethnicity [3, 9].

This study detected significant differences between regions after adjusting ASM with a GML ANOVA for all these factors and others (see Table 2). The men and women from central Mexico had significantly higher ASM values than the subjects from the northwest, even though the latter group was taller and heavier (Table 1). The independent predictors of ASM by gender in both regions were as follows: for men, age, total body mass by DXA, height, BFM, FFM, total bone mineral content, and waist circumference, while for women they were FFM, total body mass, BFM, truncal fat, and hypothyroidism. Significant differences were also found at the group level, since the older people in central Mexico had higher ASM values than their counterparts after adjusting for certain region-specific population covariates (Table 2).

In this study, the independent predictors of ASM by region were estimated total energy expenditure, truncal fat, total body mass by DXA, height, BFMI, hypothyroidism, and RMR for men and women as a group from northwestern Mexico, while truncal fat, BFMI, estimated resting metabolic rate, TBMC, and handgrip strength were the main independent predictors of ASM for older men and women subjects from central Mexico. Similar results were found when the ASMI was compared across genders and regions (Table 3).

To date, few studies have explored the influence of nonbiological variables such as demographic parameters (except age). However, only other biological covariates, such as TEE, RMR, and health status or chronic conditions on skeletal muscle component or ASMI, in addition to the traditional anthropometric and body composition measurements, were found. Recently, Tyrovolas et al. [13] published findings that suggest that lower levels of education and wealth, higher body fat mass, consumption of alcoholic beverages, and chronic conditions are all significantly associated with lower skeletal muscle indices.

The clinical relevance of the significant differences in ASMI in older people in contexts of obesity in these two regions could be that older people in some regions of the country are likely more susceptible to suffering from sarcopenic obesity. Also, the differences found in ASMI seem to counter-indicate the use of other indices, mainly those generated in different ethnic or age groups because it is now well recognized that cut-off points are important contributing factors to discrepancies in the prevalence of sarcopenia [11, 29]. In Mexico, region-specific ASMI cut-off points should be used to avoid bias in the prevalence of the aforementioned clinical entities. Additionally, our results highlight the need to define ASMI cut-off points based on young male and female adult populations in these two regions of Mexico in order to define low skeletal muscle when sarcopenia is to be defined based on -2SD of ASMI from a young reference group. It is well known that the cut-off points for Asian populations are lower than those for non-Asian subjects in both genders. The ASMI of young Asians is 15% lower than that of non-Asians; thus, using the low ASM typical of young Asians would generate lower prevalence of sarcopenia in the elderly [11]. Therefore, estimating the body composition of a younger Mexican population by gender and region will improve the accuracy of diagnoses of sarcopenia in older Mexicans.

The present study has some limitations, primarily the fact that it is a cross-sectional study that includes men and women subjects ≥60 years of age who fulfilled specific inclusion and exclusion criteria. Our sample, therefore, is not representative, so our results may be limited to these particular samples and to apparently healthy urban older people in free-living conditions, including those with some controlled diseases. The potential advantages of our study, in contrast, are that all participants were free of heart disease, physical disability, and chronic kidney disease at the time of assessment and were neither immobile nor restricted to bed rest. Subjects with these conditions and/or diseases can lose body weight and skeletal muscle mass, which would affect the main response variable of ASM. Body composition was assessed at both study sites using the same software and type and brand of densitometer, following the recommended DXA measurements protocol. Also, all images were adjusted by the same technician using the ROI appropriately to define ASM, following recommendations by Heymsfield et al. [21]. All these factors contribute to reducing the errors commonly found in multicenter studies. Finally, older people from southern Mexico, the third region mentioned at the outset, were not included in this study primarily due to the lack of the infrastructure required to measure ASM in that area. Future studies should take advantages of our results to include people from this well-characterized area of the country to explore whether our hypothesis is supported or rejected.

Despite the small sample in both regions, our anthropometric data matched quite well those published previously at the national level (ENSANUT, 2012). With respect to body composition, this is the first study to provide data on different body composition compartments and components in older people from two regions of Mexico. Our results emphasize the importance of not only taking into account ethnicity but also regionalizing data from these body composition estimates and their respective indices. Future studies should consider differences in ASM between regions when estimating the prevalence of sarcopenia, sarcopenic obesity, and osteosarcopenia using suitable, population-specific muscularity indices.

## 5. Conclusions

Older people from two regions of Mexico were found to have different anthropometry and body composition estimates. The nonadjusted mean ASM values did not differ between older people from northwestern and central Mexico, despite significant differences in body weight and height; however, when ASM was adjusted for several demographic variables, including age and other biological parameters, in addition to body weight and height, the subjects from central Mexico presented higher ASM and ASMI values despite their lower body weight and height compared to the older participants from northwestern Mexico. Our findings underscore the importance of regionalizing the ASMI when this estimate is to be used for diagnostic purposes, especially for sarcopenia, sarcopenic obesity, and osteosarcopenia. It is also important to adjust ASM or ASMI for other associated variables, in addition to the conventional ones of age, body weight, height, and gender, particularly in countries with contrasting environments like Mexico and other Latin American nations.

## Figures and Tables

**Table 1:** Age and anthropometric characteristics and body composition of older people from two regions of Mexico.

	Men		Women		Together	
Variables	Northwest	Center	Northwest	Center	Northwest	Center
	*n*=75	*n*=55	*n*=142	*n*=158	n= 217	n= 213
Age, years	72.7 ± 7.3	70.4 ± 6.7	71.2 ± 6.6	68.1 ± 5.5^*∗∗*^	71.7 ± 6.9	68.7 ± 5.9^*∗∗*^

Body weight, kg	77.1 ± 12.2	68.9 ± 7.9^*∗∗*^	68.6 ± 12.5	62.6 ± 9.8^*∗∗*^	71.5 ± 13.0	64.2 ± 9.7^*∗∗*^

Height, m	1.68 ± 0.0	1.63 ± 0.0^*∗∗*^	1.54 ±0.06	1.51 ± 0.0^*∗∗*^	1.59 ± 0.09	1.54 ± 0.0^*∗∗*^

Body mass index, kg/m^2^	27.0 ± 3.5	25.6 ± 2.4^*∗*^	28.7 ± 5.0	27.3 ± 4.1^*∗*^	28.1 ± 4.6	26.8 ± 3.8^*∗*^

Waist circumference, cm	99.1 ± 10.9	94.5 ± 6.9^*∗*^	94.9 ± 12.0	98.8 ± 10.5^*∗*^	96.3 ± 11.8	97.7 ± 9.9

Total body mass by DXA, kg	75.8 ± 12.0	68.0 ± 7.8^*∗∗*^	67.5 ± 12.3	61.9 ± 9.7^*∗∗*^	70.4 ± 12.8	63.5 ± 9.6^*∗∗*^

Body fat mass, kg	24.7 ± 7.1	18.8 ± 4.7^*∗∗*^	29.9 ± 8.1	26.1 ± 6.6^*∗∗*^	28.1 ± 8.1	24.2 ± 6.9^*∗∗*^

BFMI, kg/m^2^	8.67 ± 0.23	7.01 ± 0.27^*∗*^	12.55 ± 0.26	11.40 ± 0.25^*∗*^	11.21 ± 0.23	10.27 ± 0.23^*∗*^

Truncal fat, kg	14.5 ± 4.6	10.8 ± 2.9^*∗∗*^	15.4 ± 4.6	13.9 ± 3.9^*∗*^	15.1 ± 4.6	13.1 ± 3.9^*∗∗*^

TLT, kg	48.5 ± 6.4	47.0 ± 4.6	35.9 ± 5.1	34.3 ± 4.3^*∗*^	40.2 ± 8.2	37.6 ± 7.1^*∗∗*^

TBMC, kg	2.4 ± 0.3	2.1 ± 0.3^*∗∗*^	1.7 ± 0.2	1.5 ± 0.2^*∗∗*^	1.9 ± 0.47	1.6 ± 0.4^*∗∗*^

FFM, kg	51.1 ± 6.6	49.2 ± 4.8	37.6 ± 5.3	35.8 ± 4.5^*∗*^	42.2 ± 8.6	39.3 ± 7.4^*∗∗*^

FFMI, kg/m^2^	15.3 ± 1.9	18.3 ± 1.3^*∗∗*^	15.7 ± 1.9	16.8 ± 1.9^*∗∗*^	15.6 ± 1.9	17.2 ± 1.9^*∗∗*^

ASM, kg	19.3 ± 2.8	19.8 ± 2.2	13.5 ± 2.08	13.4 ± 1.8	15.5 ± 3.6	15.0 ± 3.4

ASMI, kg/m^2^	6.77 ± 0.007	7.39 ± 0.008^*∗∗*^	5.67 ± 0.006	5.84 ± 0.005^*∗*^	6.05 ± 0.006	6.24 ± 0.006^*∗*^

Grip strength, kg	32.5 ± 7.2	33.2 ± 7.0	18.6 ± 4.1	19.6 ± 3.9^*∗*^	23.4 ± 8.5	23.1 ± 7.7

Total body mass by DXA: total mass derived by dual energy X-ray absorptiometry; BFMI: body fat mass index; TLT: total lean tissue; TBMC: total bone mineral content; FFM: fat-free mass; FFMI: fat-free mass index; ASM: appendicular skeletal muscle mass; ASMI: appendicular skeletal muscle mass index; ^*∗*^*p* ≤ 0.05; ^*∗∗*^*p* ≤ 0.0001.

**Table 2:** Association of potential and independent predictors of ASM and their respective indices in older people by gender and region.

Dependent variables	Univariate association of potential predictors (*p* ≤ 0.2)	Independent predictors (*p* ≤ 0.05)
*ASM/region*		

Northwest	BFMI, truncal fat, total body mass by DXA, height, grip strength, TBMC, RMR, TEE, age, waist and hip circumference, family income, gender, employment status, hypothyroidism, osteoporosis, digestive problems, smoking, and alcohol	TEE, Truncal fat, Total body mass by DXA, Height, BFMI, Hypothyroidism, and RMR

Center	BFMI, truncal fat, total body mass by DXA, height, grip strength, TBMC, RMT, and TEE, gender, educational level, digestive problems, and alcohol	Truncal fat, BFMI, RMR, TBMC, and Grip strength

*ASM/gender and region *		

Men/northwest	BFM, truncal fat, total body mass by DXA, height, grip strength, TBMC, RMR, TEE, age, waist and hip circumference, educational level, and employment status	Total body mass by DXA, Height, and BFM

Men/center	TBF, FFM, truncal fat, total body mass by DXA, height, grip strength, TBMC, RMR, TEE, age, waist and hip circumference, and educational level	FFM, Height, Waist circumference, TBMC, and Age

Women/northwest	BFM, truncal fat, total body mass by DXA, height, grip strength, TBMC, RMR, TEE, age, waist and hip circumference, hypertension, hypothyroidism, osteoporosis, and digestive problems	Total body mass by DXA, BFM, Truncal fat, and hypothyroidism

Women/center	TBF, FFM, truncal fat, total body mass by DXA, grip strength, TBMC, RMR, TEE, age, and waist and hip circumference	FFM

*ASMI/region*		

Northwest	BFM, truncal fat, total body mass by DXA, height, grip strength, TBMC, RMR, TEE, waist and hip circumference, gender, employment status, hypertension, osteoporosis, digestive problems, smoking, and alcohol	TEE, Truncal Fat, Height, Total body mass by DXA, and BFM

Center	BFM, total body mass by DXA, height, grip strength, TBMC, RMR, TEE, waist and hip circumference, gender, and digestive problems	TEE, BFM, Height, Gender, Waist and hip circumference, and TBMC, and Grip strength

*ASMI/gender and region*		

Men/northwest	BFM, FFM, total body mass by DXA, height, grip strength, TBMC, RMR, TEE, age, waist and hip circumference, and educational level	FFM, Height, TEE

Men/center	FFM, total body mass by DXA, grip strength, TBMC, RMR, TEE, age, and hip circumference	FFM, Total body mass by DXA, and Waist circumference

Women/northwest	BFM, FFM, truncal fat, total body mass by DXA, TBMC, RMR, TEE, waist and hip circumference, educational level, employment status, hypertension, hypothyroidism, osteoporosis, and digestive problems	FFM, Waist circumference, Hypertension, TBMC, Hypothyroidism, and Osteoporosis

Women/center	BFM, FFM, truncal fat, total body mass by DXA, grip strength, TBMC, RMR, TEE, waist and hip circumference	FFM, TBMC, and Waist circumference

ASM: appendicular skeletal muscle mass; ASMI: appendicular skeletal muscle mass index; BFMI: body fat mass index; TBMC: total bone mineral content; RMR: rest metabolic rate; TEE: total expenditure energy; total body mass by DXA: total mass derived by dual energy X-ray absorptiometry; FFM: fat-free mass.

**Table 3:** Association of potential and independent predictors of body BFM and their respective indices in older people by gender and region.

Dependent variables	Univariate association of potential predictors (*p* ≤ 0.2)	Independent predictors (*p* ≤ 0.05)
*BFM/region*		

Northwestern	FFM, truncal fat, total body mass by DXA, height, grip strength, RMR, TEE, age, waist and hip circumference, gender, hypertension, hypothyroidism, and alcohol	Total body mass by DXA and FFM

Center	ASM, FFM, truncal fat, total body mass by DXA, height, TBMC, RMR, TEE, waist and hip circumference, and digestive problems	Truncal fat, TEE, FFM, ASM, Waist circumference, and TBMC

*BFM/gender and region *		

Men/northwestern	ASM, FFM, truncal fat, total body mass by DXA, height, TBMC, RMR, TEE, waist and hip circumference, hypertension, and digestive problems	FFM and Total body mass by DXA

Men/center	ASM, FFM, truncal fat, total body mass by DXA, height, strength, RMR, TEE, age, waist and hip circumference, and digestive problems	Truncal fat and Hip circumference

Women/northwestern	ASM, FFM, truncal fat, total body mass by DXA, height, strength TBMC, RMR, TEE, age, waist and hip circumference, osteoporosis, and smoking	Total body mass by DXA, FFM, ASM, Smoking

Women/center	ASM, FFM, truncal fat, total body mass by DXA, height, TBMC, RMR, TEE, age, waist and hip circumference, and digestive problems	Truncal fat, RMR, FFM, Waist circumference, and ASM

*BFMI/region*		

Northwestern	ASM, FFM, truncal fat, total body mass by DXA, height, strength TBMC, RMR, TEE, age, waist and hip circumference, family income, gender, employment status, hypertension, hypothyroidism, smoking, and alcohol	Hip circumference, Height, Total body mass by DXA, FFM, Strength, and ASM

Center	ASM, truncal fat, total body mass by DXA, height, strength TBMC, RMR, TEE, waist and hip circumference, gender, employment status, digestive problems, and alcohol	Truncal fat, height, TEE, ASM, Total body mass by DXA, TBMC and Waist circumference

*BFMI/gender and region*		

Men/northwestern	FFM, truncal fat, total body mass by DXA, TBMC, RMR, TEE, waist and hip circumference, hypertension, type 2 diabetes, and digestive problems	Total body mass by DXA, FFM, and Waist circumference

Men/center	FFM, truncal fat, total body mass by DXA, strength, RMR, TEE, age, waist and hip circumference, educational level, and employment status	Total body mass by DXA, Hip circumference, FFM, and Education level

Women/northwestern	ASM, FFM, truncal fat, total body mass by DXA, TBMC, RMR, TEE, age, waist and hip circumference, hypertension, and osteoporosis	Total body mass by DXA, Waist and hip circumference, FFM, Hypertension and Osteoporosis

Women/center	ASM, FFM, truncal fat, total body mass by DXA, height, TBMC, RMR, TEE, waist and hip circumference, employment status, and digestive problems	Truncal fat, Height, RMR, FFM, ASM, and Waist circumference

BFM: body fat mass; BFMI: body fat mass index; FFM: fat-free mass; ASM: appendicular skeletal muscle mass; TBMC: total bone mineral content; RMR: rest metabolic rate; TEE: total expenditure energy; total body mass by DXA: total mass derived by dual energy X-ray absorptiometry.

**Table 4:** Body fat mass and appendicular skeletal muscle mass with their respective indices in older people from two regions of Mexico.

	Men		Women		Together	
Variables	Northwestern *n* =75	Center *n* =55	Northwestern *n* =142	Center *n* =158	Northwestern *n* = 217	Center *n* = 213
Adjusted-BFM, kg	22.25 ± 0.002	22.27 ± 0.002	27.7 ± 0.007	28.1 ± 0.006^*∗*^	26.1 ± 0.005	26.3 ± 0.005

Adjusted-BFMI, kg/m^2^	7.92 ± 0.005	8.07 ± 0.0.006	11.86 ± 0.003	12.03 ± 0.003^*∗*^	10.77 ± 0.003	10.71 ± 0.003

Adjusted-ASM, kg	19.17 ± 0.10	20.16 ± 0.11^*∗∗*^	13.12 ± 0.006	13.80 ± 0.006^*∗∗*^	14.87 ± 0.006	15.79 ± 0.009^*∗∗*^

Adjusted-ASMI, kg	6.85 ± 0.003	7.28 ± 0.004^*∗∗*^	5.59 ± 0.003	5.91 ± 0.003^*∗∗*^	5.96 ± 0.002	6.33 ± 0.02^*∗∗*^

Mean values and standard error. BFM: body fat mass; BFMI: body fat mass index; ASM: appendicular skeletal muscle mass; ASMI: appendicular skeletal muscle mass index. ^*∗*^p ≤ 0.05; ^*∗∗*^p ≤ 0.0001.

## Data Availability

The dataset that supports the conclusions of this study is not readily available but can be requested for specific academic proposes.

## Abbreviations

ASM: Appendicular skeletal muscle mass
ASMI: Appendicular skeletal muscle mass index
BMI: Body mass index
BFM: Body fat mass
BFMI: Body fat mass index
FFM: Fat-free mass
TLT: Total lean tissue
TBMC: Total bone mineral content
DXA: Dual-energy X-ray absorptiometry
ROI: Region of interest
ENSANUT: National Health and Nutrition Survey
TEE: Total energy expenditure
RMR: Resting metabolic rate.
